# Improved Interhemispheric Functional Connectivity in Postpartum Depression Disorder: Associations With Individual Target-Transcranial Magnetic Stimulation Treatment Effects

**DOI:** 10.3389/fpsyt.2022.859453

**Published:** 2022-03-15

**Authors:** Yao Zhang, Yunfeng Mu, Xiang Li, Chuanzhu Sun, Xiaowei Ma, Sanzhong Li, Li Li, Zhaohui Zhang, Shun Qi

**Affiliations:** ^1^Xijing Hospital, The Air Force Military Medical University, Xi'an, China; ^2^Department of Gynecological Oncology, Shaanxi Provincial Cancer Hospital, Xi'an, China; ^3^Research Center for Brain-Inspired Intelligence, Xi'an Jiaotong University, Xi'an, China; ^4^The Key Laboratory of Biomedical Information Engineering of Ministry of Education, Institute of Health and Rehabilitation Science, School of Life Science and Technology, Xi'an Jiaotong University, Xi'an, China; ^5^The Key Laboratory of Neuro-informatics & Rehabilitation Engineering of Ministry of Civil Affairs, Xi'an, China; ^6^Xi'an Solide Brain Control Medical Technology Company, Xi'an, China; ^7^Department of Nuclear Medicine, The Second Xiangya Hospital, Central South University, Changsha, China; ^8^Department of Neurosurgery, Xijing Hospital, The Air Force Military Medical University, Xi'an, China; ^9^Center of Treatment and Rehabilitation of Severe Neurological Disorders, Xi'an International Medical Center Hospital, Xi'an, China; ^10^Henan Key Laboratory of Neurorestoratology, The First Affiliated Hospital of Xinxiang Medical University, Weihui, China; ^11^Henan Engineering Research Center of Physical Diagnostics and Treatment Technology for the Mental and Neurological Diseases, The Second Affiliated Hospital of Xinxiang Medical University, Xinxiang, China; ^12^Shaanxi Brain Modulation and Scientific Research Center, Xi'an, China

**Keywords:** postpartum depression, repetitive transcranial magnetic stimulation, voxel-mirrored homotopic connectivity, treatment effects, fMRI

## Abstract

Postpartum depression (PPD) is a depressive condition that is associated with a high risk of stressful life events, poor marital relationships, and even suicide. Neuroimaging techniques have enriched our understanding of cerebral mechanisms underlying PPD; namely, abnormalities in the amygdala-insula-frontal circuit might contribute to the pathogenesis of PPD. Stanford Accelerated Intelligent Neuromodulation Therapy (SAINT) is a recently validated neuroscience-informed accelerated intermittent theta-burst stimulation repetitive transcranial magnetic stimulation (rTMS) protocol. It has been shown to be effective, safe, tolerable, and rapid acting for treating treatment-resistant depression, and may be a valuable tool in the treatment of PPD. The purpose of the current study was to detect inter-hemispheric connectivity changes and their relationship with the clinical treatment effects of rTMS. Resting-state fMRI data from 32 patients with PPD treated with SAINT were collected and compared with findings from 32 age matched healthy controls. Voxel-mirrored homotopic connectivity (VMHC) was used to analyze the patterns of interhemispheric intrinsic functional connectivity in patients with PPD. Scores on the 17-item Hamilton Depression Rating Scale, Edinburgh Postnatal Depression Scale (EPDS) scores, and the relationships between these clinical characteristics and VMHC were the primary outcomes. Patients with PPD at baseline showed reduced VMHC in the amygdala, insula, and medial frontal gyrus compared with the HCs. These properties showed a renormalization after individualized rTMS treatment. Furthermore, increased connectivity between the left and right insula after SAINT was significantly correlated with the improvement of EPDS scores. Our results reveal the disruptions in the intrinsic functional architecture of interhemispheric communication in patients with PPD, and provide evidence for the pathophysiological mechanisms and the effects of rTMS.

## Introduction

Postpartum depression (PPD) is characterized by a series of symptoms, such as depression, agitation, and even suicide, and affects 13% of women who have just given birth (1). Psychotherapy, psychotropic medications, and electroconvulsive therapy are the primary and commonly used treatments for PPD (2, 3). Although the efficacy of these treatments has been documented, each has its limitations and shortcomings. For example, psychotherapy requires a long treatment duration and is costly (4). Women who are breastfeeding may be concerned that their infant will be exposed to psychotropic medications, and worried about the long-term developmental effects of this exposure (5). Electroconvulsive therapy is strongly recommended for the treatment of major depression, but is associated with acute adverse effects such as memory disorder and headaches (6). As such, there is an urgent need for new therapies for PPD that have minimal side effects and can be used over long durations. Repetitive transcranial magnetic stimulation (rTMS) is an effective FDA-approved treatment for major depression and is a promising treatment for PPD (7). The mechanism of rTMS in the treatment include activation of neurotransmitter systems, modulation of neural circuits and brain networks, and synaptic plasticity.

Previous studies using resting-state functional magnetic resonance imaging (fMRI) have found that patients with PPD show decreased activities in several brain regions, including the dorsolateral prefrontal cortex (DLPFC), anterior cingulate cortex, amygdala, and hippocampus, as well as attenuated cortico-cortical and cortico-limbic connectivity (8, 9). Functional network studies have also demonstrated that connectivity between the posterior cingulate cortex and right amygdala was disrupted in patients with PPD (10). Task-related fMRI studies have revealed reduced activity in the orbitofrontal cortex, dorsomedial prefrontal cortex, amygdala and striatum in patients with PPD (11). Furthermore, a diffusion tensor imaging (DTI) study found evidence of aberrant integrity of the corpus callosum, which connects the bilateral hemispheres (12). These results indicate that amygdala-insula-frontal circuit abnormality might contribute to the pathogenesis of PPD.

The DLPFC is the key TMS targeting area for treating major depressive disorder (13). Stanford Accelerated Intelligent Neuromodulation Therapy (SAINT) is an accelerated, fMRI-guided intermittent theta-burst stimulation (iTBS) protocol that has recently been shown to be effective, safe, tolerable, and rapid acting for treating treatment-resistant depression (7, 13). Whether this protocol also has promising treatment effects in patients with PPD has yet to be examined. In the current study, we applied SAINT in patients with PPD and used the voxel-mirrored homotopic connectivity (VMHC) method to investigate how SAINT influenced interhemispheric connectivity (14). We hypothesized that core regions within the amygdala-insula-frontal circuit would show normalized connectivity after SAINT protocol administration.

## Materials and Methods

### Subjects

Patients with PPD were recruited from the First Affiliated Hospital of Xinxiang Medical University. All patients were diagnosed with major depression with a puerperal onset according to the DSM-IV diagnostic criteria. No participants were receiving any pharmacological treatment. Women were excluded from the study if they had a past or current diagnosis of bipolar disorder, post-traumatic stress disorder, or other psychosis. Age matched healthy controls (HCs) were recruited from the local community. Exclusion criteria for both groups were as follows: (1) history or presence of significant neurological or medical illnesses; (2) body mass index (BMI) ≥ 30; (3) history of alcohol, drug, or smoking abuse; (4) contraindications for 3T MRI, such as claustrophobia, metal implants, and pacemakers.

### MRI Data Collection

A 3.0-T UNITED Discovery 770 MRI scanner was used for all MRI acquisitions. Participants were required to keep still and stay awake during the entire session. The resting-state functional images were obtained with the following parameters: field of view (FOV) = 224 × 224 mm, data matrix = 64 × 64, echo time (TE) = 30 ms, repetition time (TR) = 2,000 ms, slice thickness = 4 mm, flip angle = 90° and voxel size = 3.5 × 3.5 × 40 mm^3^. For anatomical reference, a high-resolution T1-weighted image was also acquired with the following parameters: TR = 7.24 ms, TE = 3.10 ms, FOV = 256 × 256 mm, flip angle = 10°, slice thickness = 0.5 mm, and and voxel size = 0.5 × 0.5 × 1 mm^3^. The same parameters were used for follow-up scans of the patients with PPD and healthy controls.

### fMRI Data Preprocessing

The resting-state fMRI images were preprocessed using the Data Processing & Analysis for Brain imaging (DPABI, http://rfmri.org/dpabi) software. The first 10 images were removed for magnetization equilibrium, and the remaining 200 images were subjected to motion realignment and slice timing, during which the mean frame-wise displacement (FD) was calculated. Subjects with more than 2 mm of maximal translation or 2° of maximal rotation were excluded. Then, the Friston-24 model was used to regress head motion effects and nuisance signals from cerebrospinal fluid white matter and head motions. Then, the fMRI data were normalized into the MNI space using the diffeomorphic anatomical registration through exponentiated lie algebra (DARTEL) method; the resulting images were finally smoothed with a Gaussian kernel of 6 mm full width at half-maximum and band-pass filtered (0.01–0.08 Hz). Before calculation of the VMHC, all preprocessed rs-fMRI data were transformed into the group-specific symmetric template; then, VMHC was computed as Pearson's correlation coefficient between each voxel's residual time series and that of the corresponding voxel in the opposite hemisphere. Subsequently, the correlation values were converted to z-values using Fisher's r-to-z transformation to enhance the normality of the values.

### Treatment

Repetitive transcranial magnetic stimulation was delivered by a commercially available magnetic stimulator (Black Dolphin Navigation Robot). Individual L-DLPFC stimulation target was determined according to a previous study (13). First, a hierarchical agglomerative clustering algorithm was applied to divide the DLPFC and subgenual anterior cingulate cortex (sgACC) into numerous functional subunits, which were defined as voxel pairs to be correlated. For each functional subunit, a single time-series value was identified, which was defined as the time-series that was most strongly correlated with the median time series. Then, Spearman's correlation coefficients were used to calculate the correlation matrix. Finally, the optimal target in DLPFC was determined considering the anticorrelation, size, spatial concentration, and dispersion of subunits. Fifty intermittent theta-burst stimulation (iTBS) sessions (1,800 pulses per session, 50-min interval) were delivered in 10 daily sessions over 5 consecutive days at 90% resting motor threshold.

### Statistical Analysis

Demographic characteristics were compared between patients with PPD and HCs using Student's *t*-tests in SPSS (IBM SPSS Statistics for Windows, version 18.0, IBM Corp.). Two-sample *t*-tests (HCs vs. patients with PPD at baseline; HCs vs. patients with PPD at follow-up) or paired *t*-tests (baseline vs. follow-up) were used to identify interhemispheric FC changes. The threshold for significance was *P* < 0.05, corrected with the FDR criterion. Age, and mean FD calculated during the preprocessing step were accounted for by including them as covariates. We extracted the mean VMHC values of the brain regions exhibiting significant differences (baseline vs. follow-up); then, Pearson's correlation coefficient was used to examine the associations between the changes in VMHC and clinical scores in SPSS. Significance was set at a threshold of *P* < 0.05, Bonferroni-corrected. Correction for multiple comparisons was accomplished using the FDR criterion with the “mafdr” script implemented in MATLAB.

## Results

### Demographic Information

All participants (patients with PPD and healthy controls) were right-handed. There were no significant differences in age, body mass index, education levels and length of pregnancy between women with PPD and HCs. As expected, the patients with PPD exhibited significantly higher EPDS scores (*P* < 0.001) and HAMD scores (*P* < 0.001) than the HCs. After rTMS treatment, all scores showed a significant improvement (*P* < 0.01 for EPDS, *P* < 0.01 for HAMD). Detailed information is listed in Table 1. The head motion indicated by mean FD did not differ significantly between baseline and follow-up in patients with PPD (*p* > 0.05; mean FD = 0.142 ± 0.035 for baseline, mean FD = 0.135 ± 0.029 for follow-up), or between patients with PPD and HCs (all *p* > 0.05; mean FD = 0.108 ± 0.041 for HCs).

**Table 1:** Demographic and clinical characteristics of participants.

**Characteristics**	**PPD (31)**	**HCs (31)**	***p***
Age (years)	31.5 ± 3.4	31.7 ± 6.3	0.91
Education (years)	13.7 ± 2.5	14.1 ± 2.9	0.65
BMI	24.3 ± 4.5	23.9 ± 4.2	0.55
Length of pregnancy(days)	281.2 ± 17.3	280.4 ± 16.9	0.76
EPDS	16.7 ± 4.6	4.75 ± 2.2	<0.01
HAMD	32.6 ± 5.2	8.64 ± 3.8	<0.01
**Characteristics**	**PI at baseline**	**PI at follow-up**	
EPDS	16.7 ± 4.6	7.88 ± 2.4	<0.01
HAMD	32.6 ± 5.2	12.1 ± 4.5	<0.01

*PPD, Postpartum depression; HCs, healthy controls; BMI, body mass index; EPDS, Edinburgh Postnatal Depression Scale; HAMD, 24-Item Hamilton Depression Scale*.

### VMHC Differences Between Groups

**Figure 1:**
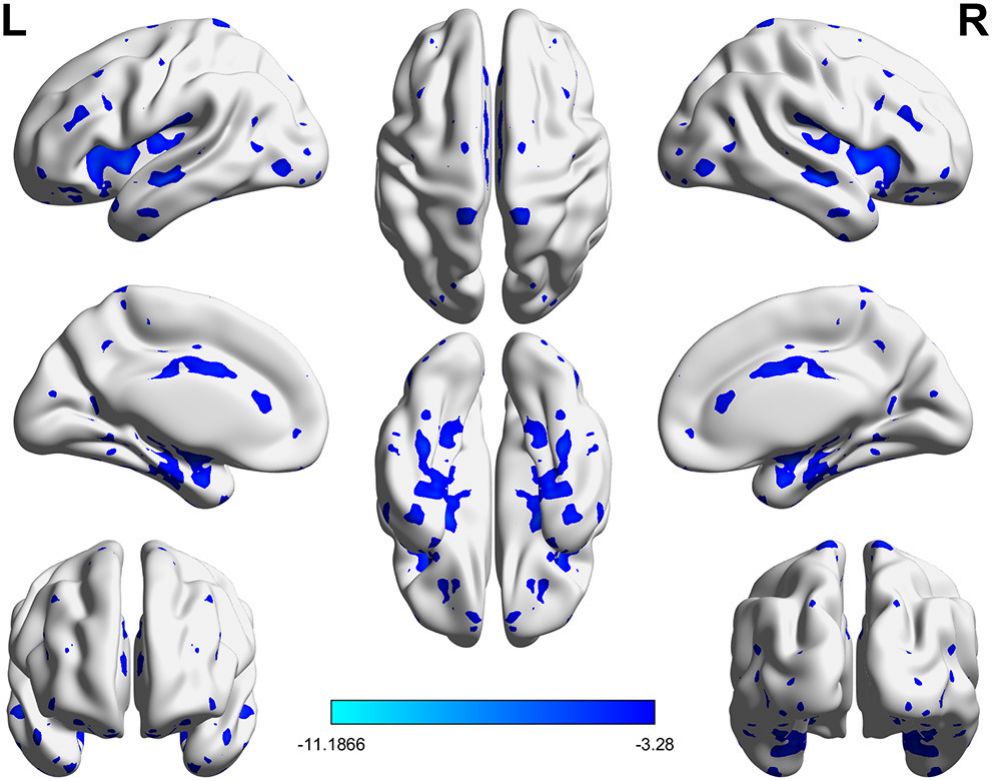
Significant differences of VMHC between PPD patients at baseline and HCs (*P* < 0.05, FDR corrected).

**Figure 2:**
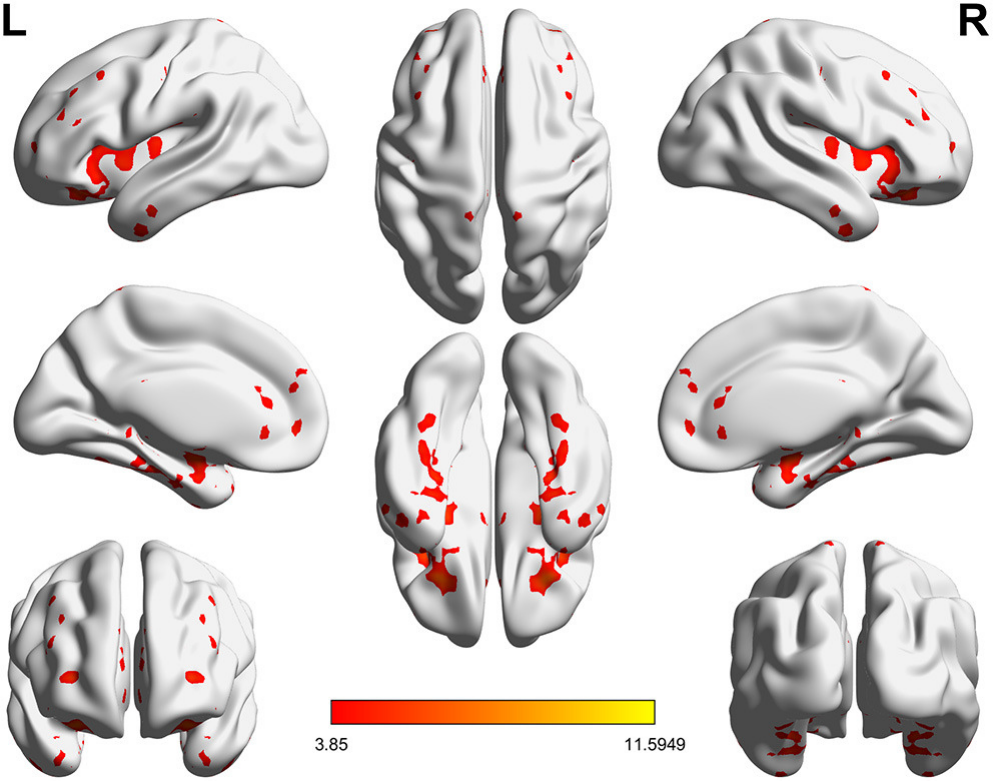
Significant differences of VMHC between PPD patients at baseline and PPD patients at follow-up (*P* < 0.05, FDR corrected).

Significant VMHC differences were found between patients with PPD and healthy controls at baseline, whereby patients with PPD showed reduced VMHC in the bilateral insula, bilateral amygdala, bilateral medial frontal gyrus, bilateral putamen, bilateral pallidum, bilateral anterior cingulate cortex, and bilateral middle cingulate cortex. After rTMS treatment, compared with baseline values, patients with PPD at follow-up showed increased VMHC in these regions, in addition, bilateral middle temporal gyrus. No significant differences were found between patients with PPD at follow-up and healthy controls. The detailed results are shown in Figures 1, 2 and Table 2.

**Table 2:** Significantly altered VMHC across the three groups.

	**voxels**	**Peak Coordinates (MNI)**			***t*** **-value**
		***x***	***y***	***z***	
**Baseline < HC** Right insula	202	42	9	0	−11.19
Left insula	202	−42	9	0	−11.19
Right amygdala	39	27	−3	−12	−7.84
Left amygdala	39	−27	−3	−12	−7.84
Right medial frontal cortex	32	24	36	−12	−4.95
Left medial frontal cortex	32	−24	36	−12	−4.95
Right putamen	152	21	21	3	−5.99
Left putamen	152	−21	21	3	−5.99
Right pallidum	40	24	−3	6	−6.01
Left pallidum	40	−24	−3	6	−6.01
Right anterior cingulate cortex	39	9	33	15	−4.39
Left anterior cingulate cortex	39	−9	33	15	−4.39
Right middle cingulate cortex	60	3	9	30	−5.21
Left middle cingulate cortex	60	−3	9	30	−5.21
**Follow-up > Baseline**					
Right insula	186	42	9	0	10.59
Left insula	186	−42	9	0	10.59
Right amygdala	30	27	−3	−12	7.15
Left amygdala	30	−27	−3	−12	7.15
Right medial frontal cortex	43	18	48	6	3.72
Left medial frontal cortex	43	−18	48	6	3.72
Right putamen	134	21	21	3	5.46
Left putamen	134	−21	21	3	5.46
Right pallidum	23	24	−6	6	4.95
Left pallidum	23	−24	−6	6	4.95
Right middle temporal gyrus	25	60	−6	−24	3.65
Left middle temporal gyrus	25	−60	−6	−24	3.65

**Figure 3:**
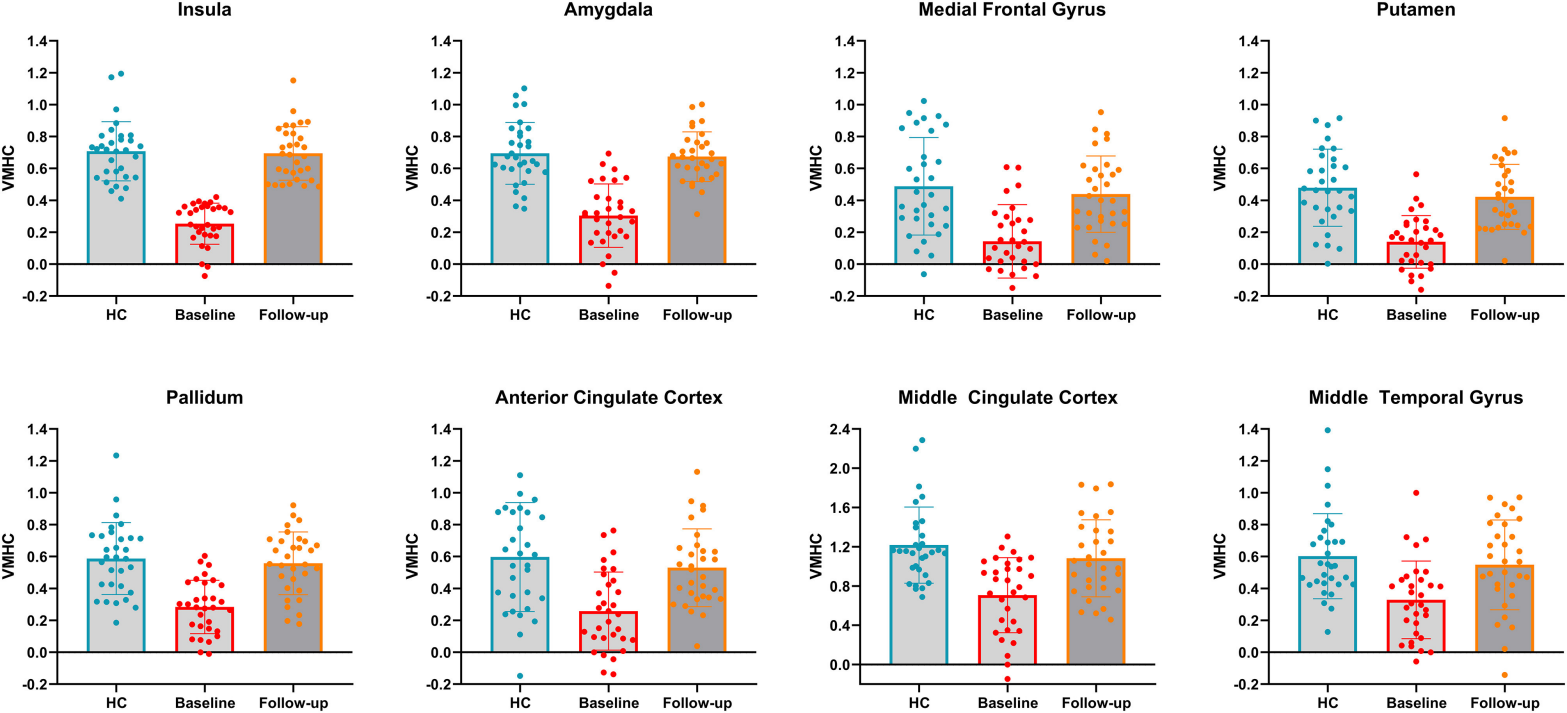
VMHC values in bilateral insula, bilateral amygdala, bilateral medial frontal gyrus, bilateral putamen, bilateral pallidum, bilateral anterior cingulate cortex, bilateral middle cingulate cortex and bilateral middle temporal gyrus across the three groups. HC, healthy controls.

To clearly demonstrate the dynamic changes in VMHC values after TMS treatment, the VMHC values within those brain regions were extracted across the three groups, as shown in Figure 3. A renormalization of VMHC changes was found in patients with PPD after TMS treatment.

### Correlation Results

**Figure 4:**
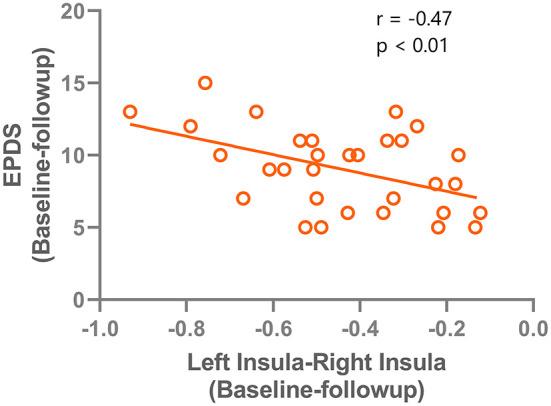
correlation results between the VMHC change (left insula - right insula) and the Edinburgh Postnatal Depression Scale improvements after rTMS treatment.

The changes of VMHC values after TMS treatment (baseline–follow-up) were extracted, and correlations with the clinical features in patients with PPD were assessed. A significant negative correlation was found between EPDS score changes and VMHC value changes in the left and right insula (*r* = −0.47, *P* < 0.001). The correlation results are shown in Figure 4. No significant correlations were found for HAMD and VMHC metrics.

## Discussion

In the current study, we found that SAINT applied to patients with PPD significantly reduced depressive symptoms. Increased inter-hemispheric connectivity was found in the amygdala-insula-frontal circuit after SAINT administration. Furthermore, the increased connectivity between the left and right insula after SAINT was significantly correlated with the improvement of the Edinburgh Postnatal Depression Scale score. Our study is the first to demonstrate that SAINT could be a promising TMS protocol for treating patients with PPD.

Multiple fMRI studies have improved our understanding of the neural mechanisms of patients with PPD. Task-related fMRI studies have indicated that during exposure to emotional stimuli, patients with PPD have increased activity in the amygdala (15) and reduced activity in the middle frontal gyrus (MFG) and inferior frontal gyrus (IFG). Resting-state fMRI studies have reported significant disruption of the posterior cingulate cortex (PCC)–right amygdala functional coupling in patients with PPD (16). Another resting-state study using regional homogeneity (ReHo) analysis found that PPD is characterized by decreased ReHo in the left DLPFC, right insular right ventral temporal cortex, amygdala, and hippocampus (17). The MFC, IFC, and PCC constitute the so-called default mode network (DMN), which is active at rest and involved in monitoring the external and internal environment (18); the right insula is a core region of the salience network (SN), which is crucial for detecting salient external stimuli and internal mental events (19). Consistent with previous studies, our findings suggest that disrupted activity within the amygdala, DMN, and SN is important for the pathophysiology of PPD.

Owing to the potential impact of medication side effects on their newborn infant or the perceived risk of breastfeeding while on medication, many mothers do not consider using psychotropic medication to treat their PPD. Compared with other depression therapies, repetitive TMS is unique in that it has no systemic side effects that would interfere with child care. Previous studies have indicated that the improvement of EPDS scores were higher in the rTMS group than the control group. As reported in previous studies, standard rTMS protocols provide marginal effects in improving the depressive mood and cognitive function of patients with PPD compared with the control group (20, 21). SAINT, however, has several advantages in improving treatment effects, such as individual DLPFC targeting, long intersession intervals to produce cumulative effects on synaptic strengthening, individualized resting motor threshold, and the use of 1,800 pulses rather than the typical 600 pulses per iTBS session.

After SAINT, increased interhemispheric connectivity was found in the amygdala, insula, and frontal gyrus, which suggests that SAINT exerts its effects by increasing inter-hemispheric communication. Interestingly, we found that the increased connectivity between the left and right insula was correlated with the improvement of depressive symptoms (indicated by a reduced Edinburgh Postnatal Depression Scale score). These findings are consistent with those of previous studies that have highlighted the importance of the salience network in the pathogenesis of depressive disorders. The salience network (SN) is involved in monitoring salient events and processing emotions (19). The deficient role of the insula might disrupt the cross-network interactions between the SN network, DMN, and limbic network, and SAINT might normalize these interactions to improve the clinical manifestations.

This study has several limitations that should be noted. First, this study had a small sample size. In the future, a larger sample size is needed to enhance the generalizability of the present findings. Second, we only explored interhemispheric functional connectivity and did not consider brain structural connectivity, other statis, or dynamic functional connectivity; examining these factors in future work will provide more important information. Third, not all PPD patients showed great improvement after SAINT administration, the underlying mechanism should be further studied in the future.

## Data Availability Statement

The raw data supporting the conclusions of this article will be made available by the authors, without undue reservation.

## Ethics Statement

Written informed consent was obtained from the individual(s) for the publication of any potentially identifiable images or data included in this article.

## Author Contributions

YZ, YM, and XM performed all data analysis and wrote the manuscript. SQ and ZZ raised the conception of the study. XL and CS contributed to the collection of MRI data. SL and LL contributed to the manuscript revision. All authors have read and approved the submitted version.

## Funding

This study was supported by the Natural Science Basic Research Program of Shaanxi (2020JQ-954).

## Conflict of Interest

XL was employed by Xi'an Solide Brain Control Medical Technology Company. The remaining authors declare that the research was conducted in the absence of any commercial or financial relationships that could be construed as a potential conflict of interest.

## Publisher's Note

All claims expressed in this article are solely those of the authors and do not necessarily represent those of their affiliated organizations, or those of the publisher, the editors and the reviewers. Any product that may be evaluated in this article, or claim that may be made by its manufacturer, is not guaranteed or endorsed by the publisher.
